# C-MYC Inhibited Ferroptosis and Promoted Immune Evasion in Ovarian Cancer Cells through NCOA4 Mediated Ferritin Autophagy

**DOI:** 10.3390/cells11244127

**Published:** 2022-12-19

**Authors:** Yanping Jin, Jianping Qiu, Xiufang Lu, Guowei Li

**Affiliations:** 1 Department of Obstetrics and Gynecology, The Affiliated Suzhou Hospital of Nanjing Medical University (Suzhou Municipal Hospital North), Suzhou 215000, China; 2 Department of Neurosurgery, The Second Affiliated Hospital of Soochow University, Suzhou 215000, China

**Keywords:** ovarian cancer, ferritin autophagy, ferroptosis, immune evasion, malignant phenotype

## Abstract

Objective: We aimed to construct the ferritin autophagy regulatory network and illustrate its mechanism in ferroptosis, TME immunity and malignant phenotypes of ovarian cancer. Methods: First, we used Western blot assays and immunohistochemistry to detect the pathway expression in ovarian cancer samples (C-MYC, NCOA4). Then, we performed RIP and FISH analysis to verify the targeted binding of these factors after which we constructed ovarian cancer cell models and detected pathway regulator expression (NCOA4). Co-localization and Western blot assays were used to detect ferritin autophagy in different experimental groups. We selected corresponding kits to assess ROS contents in ovarian cancer cells. MMP was measured using flow cytometry and mitochondrial morphology was observed through TEM. Then, we chose Clone, EdU and Transwell to evaluate the proliferation and invasion abilities of ovarian cancer cells. We used Western blot assays to measure the DAMP content in ovarian cancer cell supernatants. Finally, we constructed tumor bearing models to study the effect of the C-MYC pathway on ovarian cancer tumorigenesis and TME immune infiltration in in vivo conditions. Results: Through pathway expression detection, we confirmed that C-MYC was obviously up-regulated and NCOA4 was obviously down-regulated in ovarian cancer samples, while their expression levels were closely related to the malignancy degree of ovarian cancer. RIP, FISH and cell model detection revealed that C-MYC could down-regulate NCOA4 expression through directly targeted binding with its mRNA. Ferritin autophagy and ferroptosis detection showed that C-MYC could inhibit ferroptosis through NCOA4-mediated ferritin autophagy, thus reducing ROS and inhibiting mitophagy in ovarian cancer cells. Cell function tests showed that C-MYC could promote the proliferation and invasion of ovarian cancer cells through the NCOA4 axis. The Western blot assay revealed that C-MYC could reduce HMGB1 release in ovarian cancer cells through the NCOA4 axis. In vivo experiments showed that C-MYC could promote tumorigenesis and immune evasion in ovarian cancer cells through inhibiting HMGB1 release induced by NCOA4-mediated ferroptosis. Conclusion: According to these results, we concluded that C-MYC could down-regulate NCOA4 expression through directly targeted binding, thus inhibiting ferroptosis and promoting malignant phenotype/immune evasion in ovarian cancer cells through inhibiting ferritin autophagy.

## 1. Introduction

Ovarian cancer is a common tumor in the female reproductive system. So far, the clinical treatment of this disease has not achieved satisfactory effects, and after initial treatment, approximately 70–80% of patients suffer tumor recurrence [1,2,3]. Therefore, the development of new therapeutic targets for ovarian cancer is crucial to prevent tumor progression and improve curative effect in these cases. It has been found that iron overload could lead to excessive Fe^2+^ participating in the Fenton reaction to produce hydroxyl radicals, and if the intracellular antioxidant capacity is deficient, the hydroxyl radicals will be over-expressed and lead to oxidative stress reactions, which can produce a large number of reactive oxygen species and trigger ferroptosis. Renal carcinoma cell and leukemia cells are more sensitive to erastin-induced cell death according to the data from the National Cancer Development and Treatment Program Institute. In addition, erastin can improve the chemotherapeutic effect of temozolomide, cisplatin, cytarabine and adriamycin on specific tumors. This revealed that ferroptosis may play an important role in the inhibition of tumor growth. Previous research revealed that abnormal intracellular iron content was closely related to the occurrence of various tumors (including lung, liver and ovarian cancer), which suggested that interfering with iron metabolism during the early stages of ovarian cancer may be an effective way to kill tumor cells [4,5]. Meanwhile, relevant studies revealed that the expression of lipid reactive oxygen species in ovarian cancer cells was significantly dis-regulated compared to that in normal cells [6,7,8]. Therefore, some scholars believe that ferroptosis may play a key role in ovarian cancer progression.

C-MYC is a regulatory gene closely related to proliferation, differentiation and apoptosis of tumor cells, and its over-expression could promote the malignant phenotype of various tumors. Relative research showed that the positive rate of C-MYC in ovarian cancer tissues was 53.9%, which was higher than that in normal ovarian tissues (15.4%). Sawant et al. found that the positive rate of C-MYC was 73.1% in FIGO stage III-IV and 51.0% in FIGO stage I-II, and its expression level was closely related to the differentiation degree and clinical stage of ovarian cancer cases. Therefore, we believe that C-MYC may play an important role in the occurrence and development of ovarian cancer. Nuclear receptor coactivator 4 (NCOA4) is a selective receptor that maintains iron homeostasis by binding to ferritin, transporting it to the lysosome, and promoting autophagy degradation. It has been reported that a decrease in intracellular Fe^2+^/oxidative stress response and an increase in glutathione level are accompanied by an decrease in NCOA4 expression. Therefore, NCOA4 was considered as a positive regulator of ferroptosis. Some researchers found that the abnormal expression of C-MYC could lead to obvious changes in ferritin and Fe^2+^ content in ovarian cancer cells, while they also found that these changes were significantly correlated with NCOA4 expression [9,10,11,12,13]. Therefore, we speculate that C-MYC may affect ferritin autophagy by regulating NCOA4 expression, thus participating in the ferroptosis of ovarian cancer cells. The purpose of this study was to explore the mechanism of C-MYC in the regulation of ferroptosis and immune evasion of ovarian cancer patients through in vivo, in vitro and clinical studies, and thus provide new therapeutic targets to alleviate the chemotherapy resistance of ovarian cancer cases.

## 2. Materials and Methods

Ovarian cancer cells (SKOV3 and A2780) were provided by the Shanghai Institute of Chinese Academy of Sciences. All cells were cultured in RPMI-1640 medium containing 10% fetal bovine serum and 1% penicillin/streptomycin (Gibco, Waltham, MA, USA). Cell samples were incubated at 37 °C and 5% CO_2_, with medium changed every three days. A total of 180 clinical samples (124 ovarian cancer samples and 56 ovarian samples) were obtained in our research. The average age of patients in the ovarian cancer group was 46.19 ± 7.85 years old, with 19 cases in FIGO I stage, 38 cases in stage Ⅱ, 42 cases in stage Ⅲ, and 25 cases in stage Ⅳ. In addition, 56 patients with normal ovarian tissue diagnosed by myomectomy in our hospital in the same period were selected as the control group. The average age of patients in the control group was 49.52 ± 9.71 years old. This suggested that there was no significant difference in age distribution between these two groups (*p* > 0.05).

### 2.1. Cell Culture and Transfection

In this research, we used SKOV3 ovarian cancer cells in the C-MYC over-expression experiment group and FISH detection, while we used A2780 ovarian cancer cells in the C-MYC knock-down experiment group. To build ovarian cancer cell models with RNA/protein expression (C-MYC and NCOA4), we cloned sgRNA into the lentiCRISPR v2 vector and mixed it with the packaging plasmids psPAX2 and PMD2.G. After that, we selected ovarian cancer cells in good condition and the logarithmic growth phase and inoculated them into 6-well plates at 4 × 10^5^/well. When cell density reached 70–80%, we placed them into 1.5 mL of serum-free medium with 500 mL of a solution containing the transfection vector lipofectamine 3000 (Invitrogen, Carlsbad, CA, USA). The culture solution was replaced with complete medium after 4–6 h and then we co-transfected the plasmid into SKOV3 and A2780 cells to produce lentivirus particles. After 48 h of transfection, we collected these lentiviral particles and transfected them into SKOV3 and A2780 cells.

### 2.2. Western Blot Analysis

We selected an appropriate amount of ovarian cancer cells and clinical samples. Then, we lysed these samples on ice condition with RIPA protein lysate and protease inhibitor PMSF. After this, we proceeded with electrophoresis, mold transfer, and electro-transport, added the first antibody (1:1000), and incubated the samples at 4 °C overnight. The second antibody (1:5000) labeled with horseradish peroxidase and ECL was added in dark conditions and at room temperature before a 1-h incubation. Finally, we used Image J software (version 1.8.0) to analyze the gray value of protein bands and set GAPDH as the internal reference.

### 2.3. RNA Immunoprecipitation Assay

The RIP assay was performed using a Magna RIP™ RNA-Binding Protein Immuno-precipitation Kit, according to the manufacturer’s protocols (Millipore, Burlington, MA, USA). SKOV3 cells in the logarithmic growth phase were selected, washed with PBS, added to RIPA lysis buffer, and lysed on ice for 5 min and then the supernatant was extracted. Magnetic beads coated with AGO2 and IgG antibodies were incubated overnight at 4 °C. We obtained the protein–RNA complexes once specific proteins were captured, and all cell samples were digested with proteinase K to extract their RNA molecules. TRIzol was used to isolate RNA, which was reverse transcribed into cDNA and then we performed PCR to determine the expression of target genes. During this experiment, the magnetic beads were washed repeatedly with RIP washing buffer to remove the nonspecific adsorbent.

### 2.4. FISH Experiment

SKOV3 cells in good condition and the logarithmic growth phase were added with 6 mL of KCI solution (0.075 mol/L) preheated at 37 °C, then were placed in a constant-temperature water bath at 37 °C. We added 2 mL of stationary liquid (methanol:glacial acetic acid = 3:1) to the sample, and blew, mixed and pre-fixed the sample for 10 min at 37 °C. Then, we added 6mL of fixed liquid, and the supernatant was discarded after centrifugation; this process was performed repeatedly and the cell suspension droplets were prepared and dropped. We baked the samples at 75 °C for 30 min and then performed the FISH experiment. After hybridization, the cell samples were washed at 56 °C with 2x SSC for 5 min, added with 1% NP40 and washed for 5 min. The glass slides were taken out, dried and re-stained with 10 μL of DAPI, and finally placed under a fluorescence microscope. The results show that the nucleus displayed a blue fluorescence signal, C-MYC displayed a green fluorescence signal and NCOA4 displayed a red fluorescence signal.

### 2.5. Plasmid Transfection and Double Luciferase Gene Detection

We constructed the wild-type plasmid vector PmirGLO-h-C-MYC-WT and mutant plasmid vector using a luciferase labeling sequence (WT: ATTGAAGTGCCTTTTTGTTA, MUT: ATTGAA CACGG AATTGTTA). The day before transfection, we inoculated these SKOV3 cells into a 24-well plate (1 × 10^5^/well) with DMEM medium containing 10% FBS. Transfection operation was performed using Thermo turbofect reagent according to manufacturer’s instructions. The cells were incubated in the transfection mixture for 6 h and then replaced with complete medium. Double luciferase reporter plasmid (psiCHECK-2-C-MYC-3 ‘UTR WT and psiCHECK-2-C-MYC-3′ UTR MUT) and mRNA plasmid (NCOA4 mimic and mimic-NC) were co-transfected into SKOV3 cells in pairwise groups. The transfection groups were divided as follows: mimic-NC+psiCHECK-2-C-MYC-3 ‘UTR WT group, mimic-NC+psiCHECK-2-C-MYC-3′ UTR MUT group, NCOA4 mimic+ psiCHECK-2-C-MYC-3 ‘UTR WT group, and NCOA4 mimic+psiCHECK-2. After 48 h of transfection, the fluorescence activity was determined according to the instructions.

### 2.6. Co-Localization ANALYSIS

We selected SKOV3 cells and inoculated them into 6-well plates with a density of 1 × 10^6^/well, then we placed these cell samples in 37 °C and 5% CO_2_ condition for incubation. When cell density reached 70%, we added PFA for fixation (30 min) and 0.5% Triton X-100 for a 20 min reaction at room temperature. Goat serum was added into cell samples and sealed at room temperature for 30 min, then we discarded sealing solutions and added an appropriate amount of the primary antibody, and kept the cells at 4 °C overnight. After that, we added corresponding fluorescent probes and fluorescent-labeled secondary antibodies, and incubated them at room temperature in dark conditions for 1 h. Finally, we added DAPI and anti-fluorescence quenching agent, and placed them under a fluorescence microscope for observation.

### 2.7. Intracellular ROS Assay

All the cells were seeded in 6-well plates, and treated for 24 h according to the experimental groups and then we collected the cell precipitate. After that, we diluted the DCFH-DA fluorescent probe (2 μmol/L) or BODIPY™ 581/591 C11 fluorescent probe (7 μmol/L) with serum-free medium. The ovarian cancer cells were resuspended by adding 0.5 mL of the probe into each well and incubating at 37 °C for 30 min. The cell samples were washed with serum-free medium (twice) after shifting culture medium, then 150 μL serum-free medium was added to each sample for resuspension. At last the fluorescence intensity was detected by flow cytometry.

### 2.8. Proliferation Assay

In the colony formation assay, the transfected cells were seeded into 6-well plates and cultured in medium with 10% FBS at 37 °C and 50% CO_2_. All cells were stained with 0.1% crystal violet (BeyotimeTM, Haimen, China) after 10 days incubation, and finally colony counts were performed manually.

In the EdU assay, the transfected cells were cultured in 96-well plates and treated with 100 μL medium containing 20 μM EdU. After incubated at 37 °C and 5% CO_2_ for 2 h, all cells were fixed with 4% PA for 30 min and incubated with 0.5% Triton X-100 in PBS for 20 min, and the nuclei were stained with Hoechst for 15 min. Then, the proliferation rate was calculated according to the manufacturer’s instructions, the cells in each vision field were counted and analyzed using Image J.

### 2.9. Cell Invasion Assay

In the Transwell assay, all the samples were digested with 0.25% enzyme proteinase and centrifuged at 1200 r/min for 5 min. The number of cells added to each Transwell room was 5 × 10^5^/mL after which we placed Transwell chambers into 24-well plates to form upper and lower chambers. Then, we added 800 mL of 4% PFA to these plates and heated them for 20–30 min, washed them twice with PBS and dyed them with 0.1% crystal violet at room temperature for 30 min. Finally, the cells were observed, and photographs were taken under an inverted microscope. Cell numbers passing through the ventricular membrane were counted and analyzed.

### 2.10. Mitochondrial Membrane Potential Assay

The cells in the logarithmic growth phase were inoculated into 6-well plates with 3 × 10^5^ cells/well and treated for 24 h according to the experimental groups. Then, the mitochondrial membrane potential (MMP) was detected with JC-1 probes according to the manufacturer’s instructions (Beyotime Biotech, Haimen, China). After staining with JC-1, the cell samples were analyzed by means of flow cytometry (BD Biosciences, San Diego, CA, USA). The excitation wavelength of JC-1 was set as 488 nm, while the approximate emission wavelengths of the monomeric and J-aggregate forms were set as 529 and 590 nm, respectively.

### 2.11. Ultrastructure Observation (TEM)

SKOV3 and A2780 cells were inoculated into 6-well plates and treated for 24 h. The cells were digested using trypsin and fixed in 0.1 M phosphate buffer (pH = 7.4) supplemented with 2% PA and 2% glutaraldehyde after which we fixed these samples with 1% osmium tetroxide for 2 h. The cells were dehydrated in gradient with different concentrations of ethanol (30, 50, 70, 90 and 100%) after which the cells were co-incubated with LR white resin (Sigma, Livonia, MI, USA) for 1 h (twice) and thus embedded into this resin. The cured blocks were cut into 60 nm-diameter sections and stained with uranyl acetate and lead citrate. All cell samples were observed under a transmission electron microscope (TEM, H-7600, Hitachi High-Technologies Corporation, Yamanashi, Japan) and ultrastructure images were captured. We randomly selected vision fields and observed the morphology of the cytoplasmic nuclear membrane and the distribution of nuclear chromatin in the outer nuclear layer to average the results. Digital images were acquired by means of the AMT imaging system provided by NanJing Medical University.

### 2.12. Xenograft Study

A total of 10 BALB/c nude mice (21 days) were randomly divided into 2 groups (NC Group and si-C-MYC Group). In the NC group, we performed subcutaneous injection with 0.2 mL of SKOV3 cells (2 × 10^7^/mL) on the back of each mouse, while in the si-C-MYC group, we used 0.2 mL of C-MYC knock-out SKOV3 cells (2 × 10^7^/mL) for injection. During this process, we set the puncture point as 1.0 cm from injection site, each mouse was injected once, and the next day was marked as the first day after inoculation. We chose 6, 12, 18, 24, 30 and 36 days post tumor cell injection as the assessment points, where we measured the long/short diameter of tumors with calipers and then calculated the tumor volume according to the formula: length × width^2^/2. All the tumor bearing mice were euthanized by intraperitoneal injection with pentobarbital sodium (150–200 mg/kg) on the 40th day and then these subcutaneous transplant tumors were removed, weighed, stained and analyzed.

### 2.13. Immunohistochemistry Assay

We collected ovarian cancer tissues and para-cancerous tissues and fixed them in 10% neutral formalin for paraffin section preparation and immunohistochemical staining, Firstly, we conducted dewaxing and dehydration and then washed the dehydrated slides with distilled water to make these tissues clearer. The antigen repair was performed, and the samples were immersed in 3% H_2_O_2_ solutions for 10 min and sealed with serum, then incubated with primary antibody at a dilution of 1: 200 overnight at 4 °C. After that, we washed these slides with PBS 3 times (5 min) and incubated them with secondary antibody, added horseradish peroxidase and incubated at 37 °C for half an hour and then washed these slides with PBS 3 times. Finally, we added sample dye, dehydrated, made transparent, and sealed the samples with neutral glue, and observed them under a microscope. The immunohistochemical score was independently offered by two pathologists in double-blind detections, and C-MYC and protein showed positive staining in the cytoplasm of ovarian cancer cells (yellow): (1) according to the staining degree: no staining (0 point), light yellow (1 point), brown yellow (2 points), brown black (3 points); and (2) according to the percentage of stained cells: ≤10% (1 point), 11–50% (2 points), 51–75% (3 points), >75% (4 points). A product of staining degree and positive percentage score <3 was negative, while a product ≥3 was positive.

### 2.14. Statistical Methods

SPSS 26.0 and GraphPad Prism 8.0 were used to analyze the experimental data, and the measurement data with normal distributions were expressed as mean ± standard deviation (X ± S). Variance analysis was used to compare means among different groups, and the *t* test was used to compare means between two groups. *p* < 0.05 was considered to demonstrate statistical significance (* *p* < 0.05, ** *p* < 0.01).

## 3. Results

### 3.1. NCOA4 Pathway Showed Abnormal Expression in Ovarian Cancer Samples

**Figure 1:**
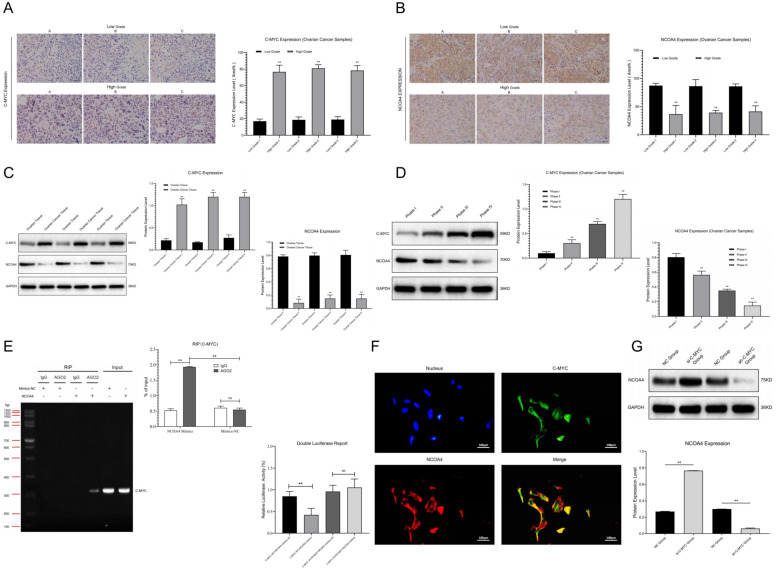
(**A**,**B**) Relative C-MYC and NCOA4 expression between low- and high-grade ovarian cancer samples were detected and analyzed by means of immunohistochemistry. (**C**,**D**) Relative C-MYC and NCOA4 expression between ovarian tissue and ovarian cancer tissue, relative C-MYC and NCOA4 expression in ovarian cancer samples with different stages (FIGO I-IV) were detected by Western blot assays and statistical analysis. (**E**) RIP and dual luciferase expression assay of pathway factors (C-MYC vs. NCOA4) and statistical analysis. (**F**) FISH analysis of co-localization between C-MYC and NCOA4. (**G**) Detection and analysis of NCOA4 expression in ovarian cancer cells after transfection. (** *p* < 0.01).

We detected the expression of signal pathways in clinical samples (Ovarian Tissue, Ovarian Cancer Tissue, FIGO I-IV) through Western blot and immunohistochemistry assays. The results showed that compared with ovarian tissues, C-MYC was significantly up-regulated while NCOA4 was obviously down-regulated in ovarian cancer samples (Figure 1C). The expression levels of C-MYC and NCOA4 were positively correlated with ovarian cancer malignancy grade (Figure 1D). The immunohistochemical staining results confirmed that compared with low-grade ovarian cancer samples, C-MYC showed obvious over-expression and NCOA4 revealed significant inhibition in high-grade ovarian cancer tissues (Figure 1A,B). All of the above results indicate that the NCOA4 regulation pathway (C-MYC/NCOA4) is closely related to ovarian cancer progression.

### 3.2. C-MYC Inhibited NCOA4 Expression through Targeted Binding

The RIP assays showed that the contents of C-MYC in NCOA4 samples treated with AGO2 were significantly higher than those treated with IgG; moreover, the contents of C-MYC in AGO2 samples treated with NCOA4 were significantly higher than those treated with mimic-NC. Double luciferase gene detection showed that compared with the NC group, the activity of C-MYC-3 ‘UTR significantly decreased after NCOA4 application in SKOV3 cells, while the activity of this region significantly increased after mutation (Figure 1E). In co-localization analysis we noticed that C-MYC (Green) and NCOA4 (Red) assumed co-localized expression in the cytoplasm of ovarian cancer cells (Figure 1F). Thus, we concluded that C-MYC could undergo targeted binding to NCOA4. Then, we transfected SKOV3 cells with plasmids containing C-MYC mimics and inhibitors for 48 h, then performed Western blot assays. The results show that the over-expression of C-MYC could inhibit NCOA4 expression, while C-MYC down-regulation could promote NCOA4 expression (Figure 1G), indicating that C-MYC might inhibit downstream NCOA4 expression through interfering with the stability of its mRNA and promoting its degradation in ovarian cancer cells.

### 3.3. C-MYC Inhibited Ferritin Autophagy and Ferroptosis through Inhibiting NCOA4 Expression in Ovarian Cancer Cells

In this section, we further investigate whether the C-MYC signal pathway could regulate ferroptosis in ovarian cancer cells. We transfected SKOV3 cells with plasmids containing RNA mimics and inhibitors (C-MYC and NCOA4) to construct ovarian cancer cell models with different pathway expression states.

**Figure 2:**
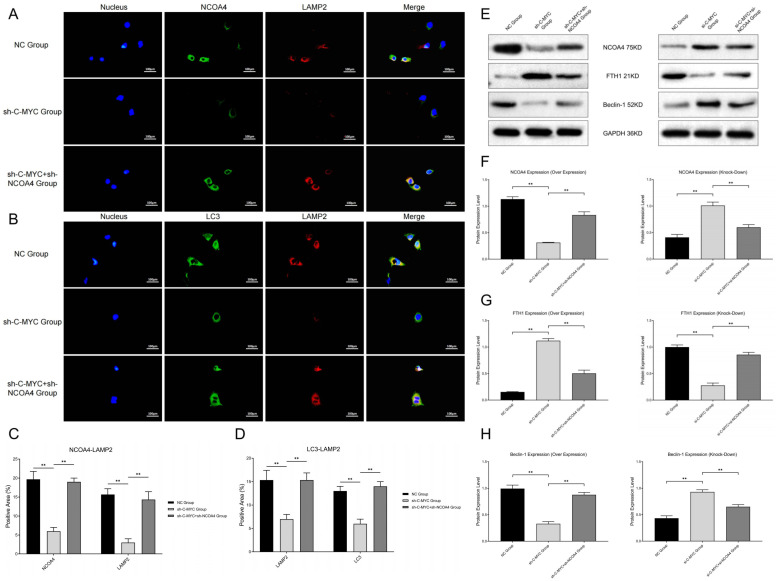
(**A**,**B**) Co-localization analysis of ferritin autophagy pathway factors (NCOA4 and LC3 vs. LAMP2). (**C**,**D**) Statistical analysis of co-localization expressions. (**E**) Western blot assays of ferritin autophagy pathway factors (NCOA4, FTH1 and Beclin-1). (**F**–**H**) Statistical analysis of ferritin autophagy pathway expression (** *p* < 0.01).

First, we detected co-localization of ferritin and lysosome labels, and the results show that the co-localization of NCOA4 and lysosome (LAMP2) was significantly inhibited in C-MYC-over-expressed ovarian cancer cells, while NCOA4 up-regulation could reverse this change (Figure 2A,C). At the same time, we also detected the co-localization of ferritin autophagy and lysosome labels, and the results show that the co-localization of the autophagy regulator (LC3) and lysosome (LAMP2) significantly decreased in C-MYC-over-expressed ovarian cancer cells, while NCOA4 up-regulation could reverse this change (Figure 2B,D). After that, we determined the ferroptosis-related index with the Western blot assay and detection kit. The results show that C-MYC up-regulation could inhibit FTH1 and Beclin-1 expression, while NCOA4 up-regulation could reverse this effect. Meanwhile, C-MYC knock-down could up-regulate FTH1 and Beclin-1 expression, while NCOA4 down-regulation could reverse this effect (Figure 2E–H). Based on the above experimental results, we concluded that C-MYC could inhibit ferritin autophagy through the NCOA4 axis in ovarian cancer cells.

**Figure 3:**
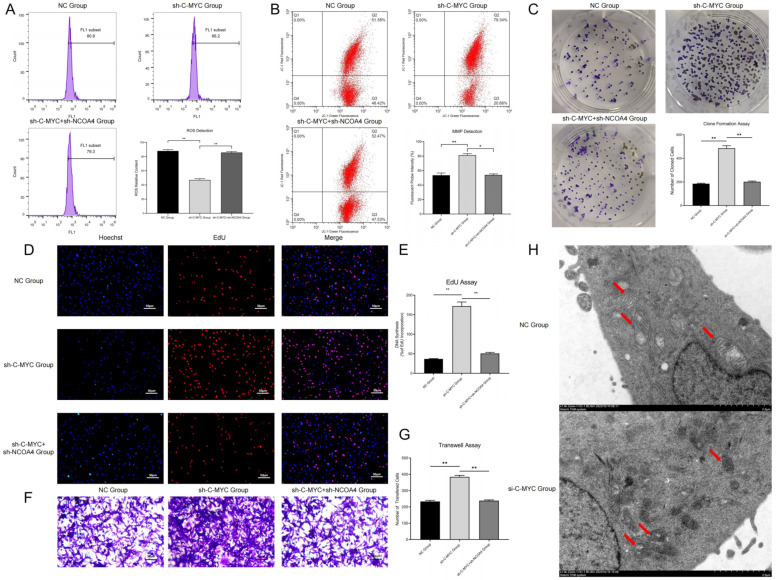
(**A**) Intracellular ROS detection in ovarian cancer cells after transfection and statistical analysis. (**B**) Detection of MMP after cell transfection and statistical analysis. (**C**) Colony formation assays of ovarian cancer cells after transfection and statistical analysis. (**D**,**E**) EdU assays of ovarian cancer cells after transfection and statistical analysis. (**F**,**G**) Transwell assays of ovarian cancer cells after transfection and statistical analysis. (**H**) Observation of mitochondrial morphology (Red Arrows) through TEM in ovarian cancer cells. (* *p* < 0.05, ** *p* < 0.01).

ROS detection showed that in C-MYC-up-regulated ovarian cancer cells, the ROS contents significantly decreased, while these changes could be reversed by NCOA4 over-expression (Figure 3A). This experimental result confirmed that C-MYC might inhibit ferroptosis in ovarian cancer cells through NCOA4-mediated ferritin autophagy.

### 3.4. C-MYC Inhibited Mitophagy and Promoted Malignant Phenotype in Ovarian Cancer Cells through the NCOA4 Axis

In this section, we used a JC-1 probe to detect the mitochondrial membrane potential in ovarian cancer cells. The results show that compared with the NC group, in C-MYC-over-expressed ovarian cancer cells, the red fluorescence intensity obviously increased, while these changes could be reversed through NCOA4 over-expression (Figure 3B). Thus, we concluded that C-MYC could maintain the stability of mitochondrial membrane potential in ovarian cancer cells through the NCOA4 axis. The observation of mitochondrial morphology (TEM) showed that compared with the NC group, in C-MYC knock-down ovarian cancer cells, the mitochondrial volume obviously reduced, the mitochondrial membrane ruptured and mitochondrial crista disappeared (Figure 3H). Thus, we demonstrated that C-MYC could inhibit mitophagy in ovarian cancer cells through the NCOA4 axis.

Colony formation assays showed that compared with the NC group, the number of cell colonies in the C-MYC over-expression group significantly increased, while this number correspondingly decreased after NCOA4 over-expression in ovarian cancer cells (Figure 3C). EdU assays revealed that the proportion of new cells in the C-MYC over-expression group obviously increased, while this proportion correspondingly decreased after NCOA4 over-expression in ovarian cancer cells (Figure 3D–E). These results indicate that C-MYC could promote cell proliferation through the NCOA4 axis in ovarian cancer cells. Transwell assays revealed that compared with the NC group, the proportion of cells passing through the basement membrane was significantly increased in C-MYC-over-expressed ovarian cancer cells, while the cell proportion correspondingly decreased after NCOA4 over-expression in ovarian cancer cells (Figure 3F–G). All of the above results indicate that C-MYC could promote a malignant phenotype through the NCOA4 axis in ovarian cancer cells.

**Figure 4:**
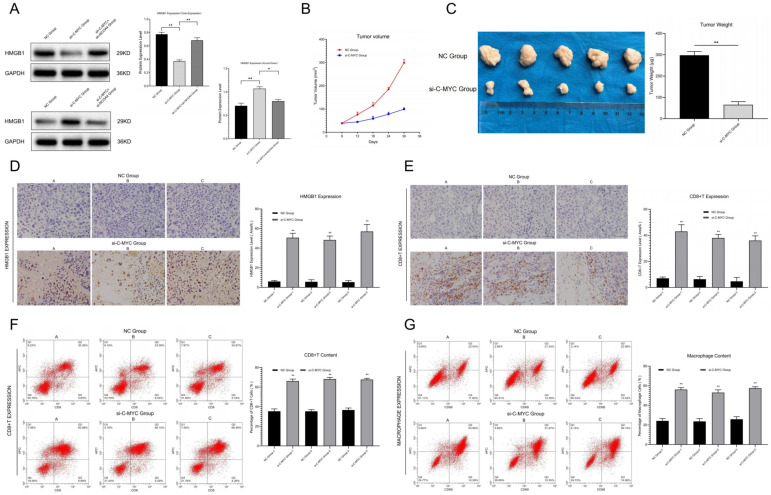
(**A**) Detection and statistical analysis of HMGB1 content in the supernatant of ovarian cancer cells after transfection. (**B**) Tumor growth curve in vivo condition. (**C**) Detection of tumor weight in in vitro condition and data analysis. (**D**) Relative HMGB1 expression between NC Group and si-C-MYC Group were detected and analyzed by means of immunohistochemistry. (**E**) Immunohistochemical staining and analysis of immune infiltration between the NC group and the si-C-MYC group (CD8+T). (**F**,**G**) Detection of CD8+T/macrophage cells through flow cytometry and statistical analysis (* *p* < 0.05, ** *p* < 0.01).

Western blot assays revealed that in C-MYC-over-expressed ovarian cancer cells, extracellular free HMGB1 content significantly decreased, while NCOA4 up-regulation could reverse this change. On the contrary, in C-MYC knock-down ovarian cancer cells, extracellular free HMGB1 content significantly increased, while NCOA4 down-regulation could reverse this effect (Figure 4A), indicating that C-MYC might inhibit the release of damage-associated molecular patterns (DAMPs) through the NCOA4 axis in ovarian cancer cells.

### 3.5. C-MYC Promoted In Vivo Tumorigenesis and Immune Evasion in Ovarian Cancer through NCOA4 Mediated Ferroptosis

The subcutaneous tumor transplantation experiment showed that compared with the NC group, the growth rate of tumors in in vivo conditions significantly slowed down (Figure 4B), while the tumor weight and volume obviously reduced (Figure 4C) in tumor-bearing mice injected with C-MYC knock-down ovarian cancer cells. The results indicate that C-MYC could inhibit the tumorigenesis of ovarian cancer cells in vivo condition.

Immunohistochemical staining and flow cytometry analysis of tumor tissues showed that in C-MYC knock-down ovarian cancer samples, the HMGB1 contents in the extracellular situation were significantly increased (Figure 4D). At the same time, microenvironment immune infiltration was significantly activated (Figure 4E–G). These results indicate that C-MYC may inhibit tumor immune infiltration in in vivo conditions through inhibiting DAMP (HMGB1) release induced by NCOA4-mediated ferritin autophagy and ferroptosis in ovarian cancer cells.

## 4. Discussion

At present, it is generally believed that ovarian cancer is one of the most dangerous threats to women’s health. Though some progress has been made in surgery and chemotherapy in recent years, approximately 75% of cases have already reached stage III–IV at the time of diagnosis, due to the lack of typical clinical symptoms in the early stage. Most scholars believed that the occurrence and progression of ovarian cancer comprise a multistage process [14,15,16]. Therefore, developing further understanding of this process’ molecular mechanisms is crucial for the diagnosis and treatment of this disease. Previous studies showed that the increased intracellular Fe^2+^ content is closely related to the occurrence of various tumors (including lung cancer, liver cancer and ovarian cancer [17]. Basuli confirmed that the Fe^2+^ level in high-grade ovarian cancer tissues was significantly higher than that in normal cases [18]. Based on this, some scholars considered that changes in Fe^2+^ content may be closely related to ovarian cancer progression.

In 2012, Dixon first defined iron-dependent, non-apoptotic cell death as “ferroptosis”, and subsequent research revealed that ferroptosis is characterized by increased Fe^2+^ and lipid peroxidation in biochemical changes. It is now believed that ferroptosis is closely related to cysteine metabolism, lipid metabolism and Fe^2+^ cycle. The inhibition of cysteine production and the reduction in glutathione are the key steps of ferroptosis, and the stages of ferroptosis include the Fenton reaction, system Xc− and GPX4 [19], which can lead to lipid peroxide accumulation and mitochondrial damage. Ferritin autophagy is a process in which ferritin is specifically recognized and transported to lysosomes for autophagic degradation, and thus releases free Fe^2+^ in normal and tumor cells. Related studies showed that the disorder of ferritin autophagy might lead to the imbalance of Fe homeostasis in cells: for example, the lack of ferritin autophagy in erythropoiesis can lead to Fe deficiency and anemia, while the over-expression of ferritin autophagy will produce large amounts of ROS and induce ferroptosis [20,21,22,23].

NCOA4 was originally described as a coactivator of multiple nuclear hormone receptors, and is closely related to the tumorigenesis and progression in ovarian cancer, prostate cancer, breast cancer and pancreatic cancer. Recent studies revealed that NCOA4 is an autophagosomes component that participates in the process of ferritin autophagy [24,25]. In 2014, Mancias confirmed that NCOA4 is a specific receptor in ferritin autophagy by proteomics, thus connecting cell autophagy with ferroptosis [26]. Subsequent studies revealed that ferritin autophagy could regulate cell ferroptosis—for example, Masaldan showed that Fe accumulation and impaired ferritin autophagy in senescent cells were closely related to the inhibition of ferroptosis [27]. Gao reported that the inhibition of NCOA4 expression could inhibit ferritin autophagy, thus inducing Fe^2+^ accumulation and lipid peroxides, which related to ferroptosis [28]. In our research, we found that NCOA4 expression was obviously down-regulated in ovarian cancer samples compared with the normal group, and its expression level was negatively correlated with the malignancy of ovarian cancer cases. Thus, we inferred that NCOA4 may play a key role in the progression of ovarian cancer, which was consistent with the results of previous studies.

Previous studies showed that NCOA4 could undergo targeted binding to FTH1 (ferritin heavy chain 1) in phagophores and transport it to the lysosome for degradation, thus releasing Fe^2+^ into the intracellular environment. Now, NCOA4 is considered as a key molecule which can promote ferroptosis in various cancers, and other studies revealed that NCOA4 down-regulation could inhibit ferroptosis by eliminating intracellular Fe^2+^, glutathione and ROS (reactive oxygen species) [29]. Most scholars consider that NCOA4 might play a critical role in cancer progression; for instance, it is thought to be involved in tumor growth and metastasis. Until now, NCOA4 has only been explored in a few cancers, and therefore it is very important to clarify the regulatory and molecular mechanisms of NCOA4 in cancer progression and thus develop innovative therapeutic approaches for these diseases. Our research confirmed that NCOA4 over-expression could promote ferritin autophagy and ferroptosis in ovarian cancer cells, while NCOA4 inhibition could achieve the opposite results; all of these results further validated previous studies.

Relevant research showed that C-MYC is an important oncogene which is located on chromosome 8q34 and can encode phosphorylated protein. It can act as a sequence-specific transcription factor which is closely related to the proliferation, differentiation, apoptosis and tumorigenesis in multiple tumors. For example, the amplification rate of C-MYC in breast cancer is approximately 30%, and it has a good predictive effect on tumor recurrence and survival. Other studies revealed that the over-expression of C-MYC can promote the progression, invasion and metastasis in ovarian cancer, and thus accelerates the malignant transformation of this tumor. Some scholars found that the abnormal expression of C-MYC in ovarian cancer cells could lead to significant changes in the expression level of NCOA4, as well as the content of ferritin and Fe^2+^ [30,31,32,33,34]. Therefore, we speculated that C-MYC may affect ferritin autophagy by regulating the expression of NCOA4 and then participate in the process of ferroptosis in ovarian cancer cells. Our research highlights that C-MYC expression is obviously up-regulated in ovarian cancer samples compared with the normal group, and its expression level is positively correlated with the malignancy of ovarian cancer cases. Thus, we inferred that C-MYC may play a key role in the progression of ovarian cancer. Meanwhile, C-MYC can undergo targeted binding with NCOA4 and inhibit ferritin autophagy and ferroptosis in ovarian cancer cells through the NCOA4 axis, which is consistent with the results of previous studies.

Some scholars believe that similar to apoptosis, ferroptosis cells may attract immune cells to its position through antigen presentation mediated by “Find Me” signal release. These cells could be swallowed by macrophages in in vitro conditions, which strongly supported this opinion. Previous studies showed that necrotic cells (such as necrosis and scorch) were characterized by a rupture in the cell membrane and the release of intracellular components, such as damage-associated molecular patterns (DAMPs, including calreticulin, ATP and HMGB1), thus attracting macrophages and improving antigen presentation efficiency. At present, it is believed that tumor cells suffering ferroptosis may also release DAMPs to the outside through autophagy progression, and thus activate and recruit immune cells. For example, in the model of myocardial ischemia/reperfusion injury, ferroptosis cells can activate immunity and recruit neutrophils by releasing DAMPs. In pancreatic cancer, tumor cells suffering ferroptosis can release KRAS, thus inducing immunosuppression and fatty acid oxidation in macrophages. Other studies showed that tumor cells with ferroptosis could release large amounts of PGE2 (prostaglandin E2), which may be related to the upregulation of prostaglandin endoperoxide synthase 2 (PTGS2). Research has confirmed that PGE2 has immune-suppressive activity and could act as an inflammatory factor. Based on the above research results, we speculated that ferroptosis tumor cells may activate the adaptive immune system by releasing specific factors (HMGB1 and PGE2) and recruiting immune cells to its position, thus achieving the elimination of tumor cells [35,36,37]. However, until now, the role of NCOA4-mediated ferritin autophagy in this process and its possible regulatory mechanisms still lack relevant research. In this experiment, we found that in ferroptosis-activated ovarian cancer cells, HMGB1 and PGE2 contents released outside the cell membrane obviously increased. At the same time, C-MYC knock-down induced ferroptosis activation, which could promote immune cell infiltration in ovarian cancer tissues. These results suggest that C-MYC/NCOA4-regulated ferroptosis is closely related to immune infiltration and immune evasion in ovarian cancer, which further clarifies the molecular mechanism by which cell death regulates the immune response in ovarian cancer.

In this research, we constructed the ferritin autophagy regulatory network in ovarian cancer through pathway detection in clinical samples. The pathway expression verification confirmed that C-MYC was significantly up-regulated and NCOA4 was significantly down-regulated in ovarian cancer samples, and their expression levels were closely related to tumor malignancy. The cell model test revealed that C-MYC could down-regulate NCOA4 expression through directly targeted binding to its mRNA, destroy its stability and promote degradation. Ferritin autophagy and ferroptosis indicator assays showed that C-MYC could inhibit ferroptosis through NCOA4-mediated ferritin autophagy, thus inhibiting ROS accumulation and mitochondrial injury in ovarian cancer cells. Functional experiments confirmed that C-MYC could promote malignant phenotypes in ovarian cancer cells through the NCOA4 axis. In vitro and in vivo studies revealed that C-MYC could inhibit DAMP release and promote immune evasion in ovarian cancer through NCOA4-mediated ferritin autophagy and ferroptosis.

## 5. Conclusions

In general, the results of this study show that C-MYC was significantly up-regulated in ovarian cancer samples and could down-regulate NCOA4 expression through directly targeted binding. This inhibited ferroptosis, promoted malignant phenotypes and motivated immune evasion in ovarian cancer through NCOA4-mediated ferritin autophagy. Our research provided new biomarkers and therapeutic targets for the clinical diagnosis and treatment of ovarian cancer patients.

## 6. Future Prospects

The binding site and regulatory mechanisms of the C-MYC/NCOA4 axis in ovarian cancer cells have not been fully clarified; we plan to expound on this point through exploration of the molecular mechanisms of NCOA4 mRNA transcription in the future. In our research, the molecular mechanism of cellular content release mediated by ferroptosis and its relationship with immune suppression and evasion in ovarian cancer have not been fully illustrated, and these subjects need to be further explored with subsequent experiments.

## Data Availability

The datasets analyzed in this study are available from the corresponding author on reasonable request.
